# Multitasking and workplace wellbeing: the roles of job stress and job autonomy

**DOI:** 10.3389/fpsyg.2025.1611876

**Published:** 2025-08-18

**Authors:** Zheng Wei, Rohayu Abdul-Ghani, Norazila Mat, Rosmah Mat Isa

**Affiliations:** Faculty of Economics and Management, Universiti Kebangsaan Malaysia, Bangi, Malaysia

**Keywords:** multitasking, workplace wellbeing, job stress, job autonomy, information technology industry in China

## Abstract

This cross-sectional study investigated how multitasking associates with workplace wellbeing, emphasizing the mediating role of job stress, and the moderating role of job autonomy. Grounded in the Job Demands-Resources (JD-R) and Conservation of Resources (COR) theories, this research proposed that multitasking negatively associates workplace wellbeing by increasing job stress, and that job autonomy buffers this relationship between multitasking and job stress. Data were collected from 354 employees in the information technology industry in China, with a response rate of 98.33%, ensuring sample adequacy. AMOS 26.0 and SPSS 29.0 were employed in this study to test the hypothesized relationships. Results indicated that job stress partially mediates the relationship between multitasking and workplace wellbeing (VAF = 35.73%). Job autonomy moderates the relationship between multitasking and job stress, weakening the adverse relationship. These findings contribute to the literature by clarifying the dual roles of job stress and job autonomy. Practically, organizations are encouraged to reduce job stressors and enhance autonomy to support employee wellbeing. Future research should explore industry-specific differences and longitudinal dynamics to deepen understanding of multitasking's impact over time.

## 1 Introduction

With the rapid advancement of digital technologies, the work environment in the Information Technology (IT) sector has undergone significant transformation. In China, the widespread adoption of advanced digital tools and platforms has made multitasking a prevalent—and often necessary—practice among IT professionals. Multitasking, defined as the simultaneous execution of multiple complex tasks, has become a common yet significant source of stress in the modern workplace (Chen et al., 2023). To enhance efficiency, employees frequently rely on various digital communication and collaboration tools to handle multiple tasks simultaneously (Yang et al., 2025). However, while digital multitasking can improve productivity under certain conditions (Crews and Russ, 2020), it also presents considerable challenges to subsequent cognitive states and behaviors of employees (Yang et al., 2025).

The phenomenon of multitasking in the digital workplace is closely tied to the concept of continuous partial attention, where individuals constantly divide their focus among multiple tasks and digital stimuli. Due to the inherent limitations of the human mind, performing multiple cognitive tasks at the same time reduces the amount of mental resources that can be allocated to each task (Mason et al., 2025). This persistent switching of attention can result in cognitive overload, mental fatigue, reduced productivity, and heightened stress levels. Moreover, in light of the rapid advancement of digital technologies, it has become increasingly important to deepen our understanding of the relationship between work activities and the lived experiences of employees within organizations (Silva et al., 2022). In recent years, especially with the rise of remote work in some organizations, these challenges have become more evident in China's IT sector. Remote work often blurs the boundaries between personal and professional life, leading to longer working hours and difficulty disconnecting from job demands. These conditions further intensify mental fatigue and negatively affect overall workplace wellbeing.

In Chinese IT industry, these challenges are further compounded by cultural and organizational factors. Take “996” work culture for example, which is characterized by working from 9 a.m. to 9 p.m., 6 days a week (72 h per week, 12 h per day), has been prevalent in many Chinese internet companies officially or informally (Zheng and Qiu, 2023). Critics say it violates China's labor laws and equate it to “modern slavery” (Wang, 2020). This cognitive overload from managing multiple simultaneous digital tasks, such as responding to instant messages, switching between development platforms, and attending virtual meetings. This working pattern has been associated with increased job stress, burnout, and negative health outcomes among employees. Additionally, the expectation of constant availability and responsiveness, known as “digital presenteeism” (Notshe, 2025), has become more pronounced in remote work environments with digital communication platforms, contributing to heightened job stress and reduced wellbeing employees. This environment necessitates a deeper understanding of how multitasking, job stress, and job autonomy are related to workplace wellbeing.

The relationship of multitasking with both employees and organizations has been studied in some existing studies, yet conclusions remain inconclusive and context-dependent. Without a comprehensive grasp of its implications, there is a risk that multitasking would continue as a standard workplace practice without adequately addressing its complexities (Pluut et al., 2024). Multitasking is theoretically considered to have both advantages and disadvantages. On one hand, it enables employees to manage diverse responsibilities and meet workplace expectations and even positively effect on subsequent creativity (Kapadia and Melwani, 2021). On the other hand, multitasking leads to increasing job stress (Ghazanfar and Jumani, 2021), particularly when employees face excessive demands or insufficient resources to cope with task-switching. Furthermore, the stress of multitasking can lead to several psychological states and behaviors, such as anxiety (Spink et al., 2008), role ambiguity (Adler and Benbunan-Fich, 2015), role overload (Wetherell and Carter, 2014), and reduce creative engagement (Yang et al., 2025).

Employees emphasize meaningful experiences and personal wellbeing. In the era of digital transformation and high-efficiency demands, management should adopt a humanistic approach, focusing on employees' physical and mental health. Employees wellbeing consists of three aspects: life wellbeing, workplace wellbeing, and psychological wellbeing (Zheng et al., 2015). Among them, workplace wellbeing includes more than just job satisfaction; it also involves the positive feelings a person experiences in relation to their work (Zheng et al., 2015). While IT organizations in China face increasing pressure, exploring ways to enhance or maintain workplace wellbeing of employees is vital and meaningful for organizational success in today's working environment (Ji and Xu, 2023).

In this study, job stress serves as a critical mediator in the connection between multitasking and workplace wellbeing. Multitasking often introduces time pressure, frequent interruptions, and cognitive strain, all of which contribute to elevated stress levels. In turn, job stress is consistently associated with adverse results, for example emotional exhaustion (Chênevert et al., 2021; Xu and Wang, 2023), reduced life satisfaction (Kim et al., 2023; Kismono et al., 2023), and impaired health (Jeong et al., 2024; Shi et al., 2022). In IT organizations, the demands of handling multiple digital tasks of employees simultaneously can lead to increased stress levels, which in turn negatively impact overall wellbeing. Therefore, understanding this mediating mechanism is essential to untangling how multitasking translates into workplace wellbeing outcomes.

Job autonomy, refers to employees' control over how and when they perform their tasks, is a valuable resource that enhances intrinsic motivation (Langfred and Moye, 2004), strengthens self-efficacy, and fosters work enthusiasm. Drawing on Conservation of Resources Theory (Hobfoll, 2001), when individuals experience resource depletion due to multitasking, if access to additional resources—such as job autonomy—can buffer the negative impacts of stress. By allowing employees to prioritize tasks and manage their workloads more effectively, job autonomy helps alleviate the cognitive overload associated with multitasking. This, in turn, reduces job-related stress, enhances employees' capacity to cope with multitasking demands, and supports overall workplace wellbeing. This study proposes that job autonomy acts as a compensatory resource, mitigating the adverse relationship between multitasking and job stress and ultimately promoting employee workplace wellbeing. Understanding this dynamic is crucial for organizations aiming to design roles and work environments that support both performance and psychological health of employees.

Based on the existing literature, several research gaps have been identified in the study. Despite the extensive body of literature on multitasking and employees related consequences (Kapadia and Melwani, 2021; Pluut et al., 2024; Yang et al., 2025), few studies have simultaneously examined the mediating and moderating psychological mechanisms that explain how multitasking is associated with employee workplace wellbeing. Moreover, the combination of the Job Demands-Resources (JD-R) model and Conservation of Resources (COR) have been applied in occupational health research (e.g., Lackey et al., 2025; Seo et al., 2025), yet its application in the context of China's IT industry is limited. Applying this framework to study the interplay between multitasking, job stress, job autonomy, and workplace wellbeing in this specific cultural and organizational setting could yield valuable insights. Furthermore, most empirical research in this area has been conducted in western contexts, leaving limited understanding of how these relationships manifest in culturally distinct environments, such as China's high-pressure IT sector.

To address these gaps, the present study draws on the Conservation of Resources (COR) theory (Hobfoll, 2001) and the Job Demands-Resources (JD-R) model (Bakker and Demerouti, 2017) to investigate how multitasking contributes to job stress and workplace wellbeing. Within this framework, job autonomy functions as a key personal resource that can buffer the negative association of multitasking by reducing stress and preserving wellbeing. By examining these mechanisms within the specific context of the Chinese IT industry—where multitasking is widespread, job autonomy often constrained, and job stress levels is high—this study contributes to the cross-cultural validation and theoretical boundary testing of widely accepted occupational stress models and informs strategies to foster healthier, more sustainable work environments in the digital era.

This study contributes by applying the COR and JD-R models within a culturally specific context, thereby extending theoretical boundaries. It also integrates both the mediator job stress and the moderator job autonomy in a single framework—an approach rarely used in multitasking research. Furthermore, the study advances theory by exploring whether job autonomy can buffer the association of multitasking stress under conditions of high-tech pressure and hierarchical work culture.

## 2 Literature review

### 2.1 Theoretical framework

This study integrates the Job Demands-Resources (JD-R) model and the Conservation of Resources (COR) theory to provide a complementary theoretical foundation for understanding the mechanism through which multitasking affects workplace wellbeing—via job stress as a mediator, and job autonomy as a moderator.

The JD-R model (Bakker and Demerouti, 2017) serves as the structural foundation of the framework of this research. It conceptualizes multitasking as a job demand that exhausts individuals' physical and psychological resources, leading to strain (job stress) and ultimately reducing employee wellbeing. Simultaneously, it positions job autonomy as a job resource that can buffer the impact of demands on strain, thus highlighting both mediating and moderating pathways.

To further enrich this structural view, we draw on COR theory (Hobfoll, 1989, 2001) to explain the underlying psychological processes. COR posits that individuals strive to conserve and protect their valued resources. Multitasking, from this perspective, accelerates resource depletion through constant attention switching and cognitive overload. Job stress is seen as a signal of actual or threatened resource loss. In contrast, job autonomy acts as a contextual resource that enables individuals to regain control and replenish resources, thereby moderating the relationship between multitasking and job stress.

In summary, JD-R clarifies the structure of the relationships (demand → stress → wellbeing, with resource moderation), while COR provides insight into the motivational and coping mechanisms behind these effects. The integration of the two theories strengthens the conceptual logic of our model and supports both the mediation and moderation hypotheses tested in this study.

#### 2.1.1 Multitasking

Multitasking involves handling two or more cognitive or information-processing tasks at the same time (Kirschner and De Bruyckere, 2017), similar to what Ruthruff et al. (2003) describe as dual-tasking with equal attention. However, because the human brain operates like a single-core processor, it cannot truly perform tasks simultaneously. Instead, it rapidly switches between them—a process Salvucci and Taatgen (2008) term “threaded cognition,” where different cognitive resources are alternated (Kirschner and De Bruyckere, 2017).

At the individual level, multitasking requires allocating limited cognitive resources across several tasks (Spink et al., 2008). Performance is usually influenced by task complexity, processes, and available resources (Spink et al., 2008). In the workplace, multitasking often arises from the need to manage multiple subtasks within a limited timeframe. Due to time pressures and cognitive limits, tasks are often paused and resumed later, making effective switching essential (Ghazanfar and Jumani, 2021).

Multitasking in the workplace is not a uniform cognitive process; its demands and consequences vary significantly depending on the cognitive level required by the tasks involved. Rasmussen's (1983) framework of skill-based, rule-based, and knowledge-based behavior provides a valuable lens through which to interpret these differences. Skill-based behavior involves routine, automatic actions with minimal conscious effort—such as typing or navigating familiar software—which are typically resilient to multitasking interference. In contrast, rule-based behavior draws on learned procedures or heuristics and is more sensitive to task-switching, particularly when conflicting rules are activated. Most cognitively demanding is knowledge-based behavior, which requires conscious reasoning, problem-solving, and mental modeling. When multitasking involves two or more tasks that both demand knowledge-based control, performance deteriorates markedly due to shared attentional and working memory resources. Therefore, simultaneous execution is more feasible when one task is skill-based and the other is rule-based, but becomes error-prone and stressful when both are knowledge-based. This distinction is critical for understanding why multitasking is especially problematic in high-cognitive-load environments such as the IT sector, where employees must frequently switch between complex problem-solving tasks under time pressure.

Multitasking is especially prevalent in digital and remote work contexts where employees must rapidly switch between communication tools, software platforms, and concurrent work responsibilities. This is particularly relevant for IT employees who work in environments rich in cognitive demands and digital interactions.

Multitasking in the workplace is a double-edged sword. While often linked to increased productivity, especially in digital and remote settings, it carries notable cognitive and performance costs. In IT and high-demand environments, employees frequently switch between tools and tasks, creating both opportunities and challenges. Offer and Schneider (2011) note that while multitasking may raise output, switching between complex tasks increases errors and wastes time. Nearly 25% of interrupted tasks were not resumed the same day. When resumed, it took over 25 min, often after switching through at least two other tasks (Mark et al., 2005). Routine tasks may be managed concurrently, but focus-intensive work suffers significantly (Silva et al., 2022). Salvucci and Taatgen (2010) found that attention spans last just 3 min before switching, often driven by both internal and external cues—adding to mental strain.

Research links multitasking with fatigue, stress, and lower accuracy. Physiological responses include increased heart rate and mental strain (Paridon and Kaufmann, 2010). Workers report reduced focus and performance (Mark et al., 2016). Simultaneously managing communication channels increases perceived stress and time pressure (Su and Mark, 2008).

Multitasking relies on intricate cognitive processes such as goal management, prioritization, and the distribution of attention. These processes engage both working memory and long-term memory (Silva et al., 2022). Posner (1980) distinguishes between automatic and voluntary attention switching, highlighting individual differences in multitasking abilities. Some employees naturally prefer sequential task completion (Kirchberg et al., 2015), while others manage multiple tasks simultaneously.

Performance efficiency is another critical concern. Rekart (2011) argues that multitasking splits attention, impairing both immediate learning and the ability to retain information over the long term. It was established that multitasking takes more time to complete tasks and negatively impacts creativity (Marchewka et al., 2020). Furthermore, structured guidelines and the proper integration of technology can help alleviate some negative links, potentially transforming multitasking into a more effective strategy under the right conditions (Ghazanfar and Jumani, 2021).

Peifer and Zipp (2019) found that multitasking lowers “flow”—a short-term peak experience of full immersion in an activity—which may be a key mechanism linking multitasking to reduced job performance (Pluut et al., 2024). Sanderson et al. (2013) found that multitasking ability improves performance only among those who prefer it. Without that preference, multitasking skills don't help.

Multitasking weakens attention and encoding, limiting what people retain in short-term memory (Butt and Warraich, 2022). Solutions include better system design and automation. Hickey and Collins (2017), and Ruscio et al. (2018) suggested replacing manual processes with technology to reduce overload. Wang et al. (2020) used a Timed Petri Nets (TPN) model to show how task complexity affects multitasking. Liu and Li (2012) added that experience helps people handle complex multitasking better.

While multitasking is a pervasive feature of modern digital work, its association with wellbeing are nuanced and highly context-dependent. Although it offers potential productivity benefits, it also introduces substantial cognitive strain and stress. These challenges are especially relevant in remote, digitally intensive environments like those faced by Chinese IT workers. A focused study on this population can help address key theoretical and practical gaps in the literature. To mitigate the negative links and enhance performance, effective multitasking management strategies—tailored to individual capabilities and supported by technology—are essential.

#### 2.1.2 Workplace wellbeing

In recent years, employee wellbeing has become a key focus in organizational research, as it's closely tied to performance and long-term success (He and Guo, 2025). In Chinese culture, wellbeing includes three areas: workplace, life, and psychological wellbeing (Zheng et al., 2015). Workplace wellbeing is a critical aspect of subjective wellbeing in professional settings, influencing employee welfare and long-term organizational sustainability (Murat et al., 2011). It is defined as a sense of prosperity from work, shaped by both intrinsic and extrinsic work values and workers' core affect (Anwarsyah et al., 2012; Aryanti et al., 2020). When employees feel well at work, they tend to be healthier and more productive, which benefits the organization (Aryanti et al., 2020).

Given that employees spend a significant portion of their lives at work—with links that extend into personal life—poor wellbeing can lead to lower productivity, poor decision-making, and increased absenteeism (Aryanti et al., 2020; De Simone, 2014). Conversely, high workplace wellbeing enhances psychological capital (hope, self-efficacy, resilience, and optimism) and strengthens organizational commitment and performance. Factors like demographic characteristics, personality traits, organizational climate, and job demands also play essential roles in shaping workplace wellbeing (Burns and Machin, 2013).

Since 2017, there has been a notable surge in publications addressing wellbeing, reflecting a growing emphasis on psychological and emotional dimensions (Judijanto, 2024). Literature shows that both external and internal factors are associated with workplace wellbeing (Imaduddin, 2024). External elements such as leadership style, job demands, and workplace culture play a critical role, but so do individual psychological traits. Employees' personal attributes—such as optimism, communication skills, emotional regulation, and social-emotional intelligence—can significantly enhance their sense of wellbeing at work. This suggests that wellbeing can be fostered not only through top-down organizational initiatives but also through personal development and bottom-up engagement (Biggio and Cortese, 2013).

Workplace wellbeing is strongly linked to key organizational outcomes. Positive co-worker relationships also boost productivity and wellbeing (Imaduddin, 2024). Technology now plays a major role, with digital tools like mental health apps, AI surveys, and wellness platforms becoming central to wellbeing efforts (Judijanto, 2024). Companies are also using broader strategies—such as flexible work, financial programs, ergonomic design, nutrition, and inclusion—to support employees. These efforts help attract, retain, and motivate talent in today's competitive market (Varis et al., 2024).

Research on workplace wellbeing is shifting from a reactive model focused on preventing harm to a proactive model cantered on promoting holistic employee flourishing. Future directions may be more emphasize personalization, digital innovation, and inclusive practices that connect individual fulfillment with organizational success.

#### 2.1.3 Job stress

Job stress is a coping reaction activated by certain psychological or physiological mechanisms when external situations or pressures place demands on an individual. Although stress is generally seen in a negative light, its association can be beneficial at low to moderate levels by boosting performance, while high stress can severely impair it. Workers under high stress may show physiological symptoms, though this is not always the case. Essentially, stress occurs when an individual's behavior exceeds their physical or mental capacity due to either external pressures or internal processes. It is a reaction—both physical and mental—to environmental changes perceived as disruptive or threatening, often linked to job roles, organizational structure, and interpersonal relationships. Excessive stress can result in adverse outcomes such as absenteeism, workplace accidents, and injuries (Ghazanfar and Jumani, 2021).

#### 2.1.4 Job autonomy

Job autonomy refers to the degree of freedom and independence employees have in carrying out their tasks (Morris and Feldman, 1996). It has been widely recognized as a key element in some work-related theories (Jang and Kim, 2025). In Job Characteristics Theory (Oldham et al., 1976), autonomy is one of the five core job features, alongside skill variety, task identity, task significance, and feedback. Similarly, Job Demand-Control Theory (Karasek, 1979) emphasizes the value of autonomy in managing work stress (Jang and Kim, 2025).

Job autonomy reflects organizational trust and serves as a key support resource that helps reduce the impact of work stress and demands (Guo et al., 2025). According to Conservation of Resources Theory (Hobfoll, 1989), high demands drain employee resources, leading to fatigue and exhaustion (Luqman et al., 2021; Zhang and Mao, 2025). As a motivational factor, job autonomy helps balance resource use and reduces burnout (Pak et al., 2023). It also moderates the association of long work hours with job satisfaction (Dong et al., 2023) and supports resource growth and resilience, promoting wellbeing and reducing negative outcomes (Guo et al., 2025).

Job autonomy has been consistently linked with positive job outcomes such as satisfaction, motivation, and performance. For example, autonomy significantly improves job satisfaction and is associated with better job performance, though it may not necessarily reduce job stress unless paired with self-efficacy or other support mechanisms (Saragih, 2011). Similarly, autonomy was shown to enhance engagement, particularly during periods of external uncertainty, such as economic turbulence, when organizations benefit from allowing employees more control over their work processes (Kidane and Xuefeng, 2021).

In addition, autonomy's effectiveness may depend on moderating variables such as job tenure (Denton and Kleiman, 2001) and personality traits (Mack, 2000). For instance, long-tenured employees or those with high conscientiousness are more likely to benefit from autonomy, indicating that one-size-fits-all empowerment strategies may not be universally effective (Denton and Kleiman, 2001).

Finally, the existing studies underscore the multifaceted nature of job autonomy and its significance for both individual and organizational outcomes (Khoshnaw and Alavi, 2020). Scholars are calling for a more nuanced and multidimensional approach to studying job autonomy, especially in light of evolving workplace structures and technologies. Future research is encouraged to explore autonomy's role in remote work, hybrid models, and AI-augmented job environments, as well as to consider the socio-cultural and organizational contexts that enable or constrain its implementation (Weber et al., 2020).

### 2.2 Hypothesis development

#### 2.2.1 Multitask and workplace wellbeing

In the fast-paced digital work environments typical of IT sectors, especially in countries like China where long working hours and high productivity expectations are common, multitasking has become a pervasive norm. Multitasking, while often viewed as a necessary skill in modern work environments (Howard and Cogswell, 2023), can lead to adverse outcomes for employee wellbeing at work due to its demanding nature.

The Conservation of Resources (COR) Theory (Hobfoll, 1989) suggests that people are motivated to acquire, preserve, and safeguard their resources, including time, energy, and emotional wellbeing. Multitasking depletes these resources by requiring continuous effort and rapid task-switching. When employees are unable to replenish their resources, they experience stress and a decline in wellbeing (Westman et al., 2004). The persistent demand to juggle multiple tasks can lead to a sense of resource loss, resulting in reduced job satisfaction, emotional exhaustion, and lower overall wellbeing.

Existing studies demonstrate the negative relationship between attentional controls for multitasking and wellbeing at work (Silva et al., 2022). Research (Xu et al., 2021) indicated that high-interactive multitasking, whether in-person or technology-mediated, consumes more cognitive resources and disrupts work more than low-interactive multitasking. According to Wang et al. (2015), it involves greater social engagement, behavioral responses, reduced task-switching control, and higher emotional involvement, leading to greater work interference. Individuals who frequently multitask at work experience decreased life satisfaction and higher rates of psychological distress. The inability to focus on a single task for extended periods disrupts workflow, reduces feelings of accomplishment, and diminishes overall workplace wellbeing.

Multitasking can also impact employees' psychological wellbeing (Xu and Wang, 2021) by contributing to feelings of being overwhelmed and a lack of control over their work environment. The constant need to switch tasks can create a sense of unfinished work and perpetual time pressure, which undermines employees' ability to experience positive emotions and job fulfillment. The emotional toll of managing multiple competing demands can lead to anxiety and depression, further reducing workplace wellbeing.

Multitasking negatively impacts workplace wellbeing, as supported by Conservation of Resources (COR) Theory (Hobfoll, 1989) and empirical studies. It increases cognitive and emotional strain, leading to higher job stress. This effect is intensified among IT employees in China due to high digital demands and cultural expectations. The findings highlight the need for organizational strategies to reduce multitasking and enhance employee resources to support wellbeing. The above analysis led to the following hypothesis.

H1: There is a negative and significant relationship between multitasking and workplace wellbeing.

#### 2.2.2 Mediation role of job stress

The COR theory (Hobfoll, 1989) suggests people aim to gain, protect, and keep valuable resources like time, energy, and wellbeing. When job demands exceed these resources, stress, and poor wellbeing follow (Mukti et al., 2021). Multitasking splits attention, causes cognitive overload, and drains energy. Without recovery, this constant resource loss leads to stress (Baka, 2015). High multitasking demands make it harder for employees to preserve their resources, increasing job stress. In tech-heavy roles like IT jobs in China, multitasking is common due to real-time communication, tight deadlines, and agile work methods. Frequent task-switching breaks focus, raises mental load, and disrupts work flow—key triggers of stress.

Job stress plays a key role between multitasking and workplace wellbeing, reflecting the mental and emotional drain caused by high task demands. Based on COR theory, employees try to protect and restore resources, but constant multitasking and limited recovery lead to ongoing stress. This stress harms mental health—causing burnout, exhaustion, and low engagement—and also affects physical health, leading to issues like fatigue, headaches, and sleep problems, which can worsen over time (Spink et al., 2008; Zampetakis and Gkorezis, 2023).

Job stress mediates the link between workplace demands and negative outcomes like poor wellbeing and job dissatisfaction. The more stress employees face from multitasking, the more their wellbeing declines. If they can't recover lost resources, prolonged stress leads to emotional burnout and disengagement. While COR theory suggests employees seek support or rest to cope, if these are lacking, their wellbeing continues to suffer. In China's IT sector, prolonged exposure to stress often goes unaddressed due to stigma around mental health and limited organizational support, exacerbating the negative consequences. Therefore, job stress acts as a critical psychological mechanism through which multitasking affects employee workplace wellbeing.

In China's digital IT work environment, frequent multitasking and limited stress management resources raise job stress levels, lowering workplace wellbeing. Supported by theory and research, this suggests job stress mediates the link between multitasking and workplace wellbeing. Recognizing this helps organizations develop stress management strategies to protect employee health and performance. Based on this, the following hypothesis is proposed.

H2: Job stress mediates the relationship between multitasking and workplace wellbeing.

#### 2.2.3 Job autonomy as a moderation variable

The JD-R model (Bakker and Demerouti, 2007) provides a strong theoretical foundation for understanding how job autonomy moderates the relationship between multitasking and job stress. According to the JD-R model, work characteristics can be classified into job demands (e.g., multitasking) and job resources (e.g., job autonomy). While high job demands can lead to stress, job resources can buffer this negative relationship, reducing stress and promoting employee wellbeing (Vui-Yee and Yen-Hwa, 2020).

Multitasking requires employees to switch between multiple tasks, leading to cognitive overload and time pressure, both of which increase job stress. Research shows that employees experiencing working demands without adequate resources (such as autonomy) are more prone to stress and burnout (Gazali et al., 2021).

Job autonomy gives employees the ability to manage their work processes, schedules, and decisions, helping to lessen the adverse links of multitasking. For IT employees in China, where multitasking is often embedded in day-to-day workflows due to agile project cycles, constant digital communication, and performance-driven culture. According to the JD-R model, job resources support employees in handling job demands by lowering the mental and physical toll of stress. Empirical studies confirm that autonomy moderates the relationship between work demands and stress, with employees experiencing lower stress levels when they have greater autonomy (Wallgren, 2013).

Vui-Yee and Yen-Hwa (2020) found that employees with high job autonomy experienced weaker relationships between workplace stressors and negative job outcomes, demonstrating the buffering role of autonomy. Gazali et al. (2021) reported that job autonomy reduces the negative impact of workload and multitasking on stress, as employees can adjust their work strategies to manage competing demands effectively. Wallgren (2013) found that IT consultants with low autonomy and high job demands had four times higher stress levels than those with higher autonomy, reinforcing the JD-R model's buffering hypothesis.

In China's hierarchical tech industry, low job autonomy can increase the stress caused by multitasking. Without control over tasks or schedules, demands feel imposed, raising stress due to lack of control. In contrast, high autonomy—such as flexible scheduling or managing digital boundaries—helps IT employees manage multitasking, reduce interruptions, and maintain control, easing stress. Thus, in digital, high-pressure IT environments, autonomy is expected to moderate the multitasking–stress link: high autonomy weakens the impact, while low autonomy intensifies it. Based on the above discussion, it proposed the following research hypothesis:

H3: Job autonomy is expected to moderate the relationship between multitasking and job stress, such that the negative effects of multitasking on job stress are weaker when job autonomy is higher.

### 2.3 Research framework

**Figure 1:**
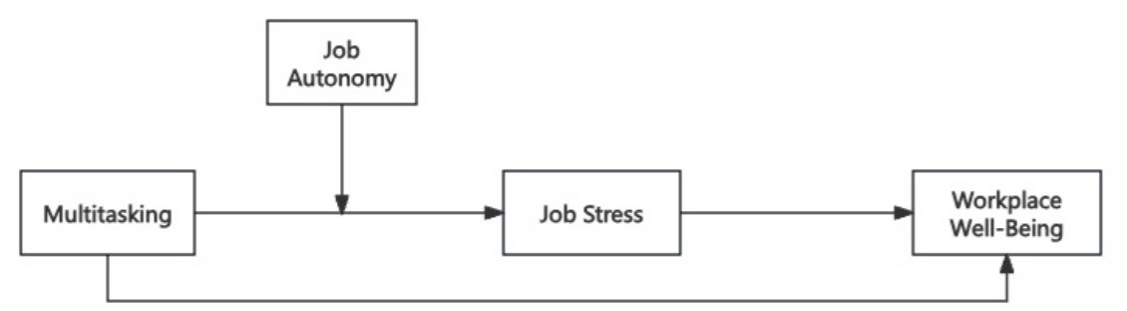
Research framework.

Figure 1 summarizes the proposed research model and illustrates the hypothesized relationships among the study variables.

## 3 Methodology

To examine the validity of the proposed hypotheses and determine the soundness of the theoretical model, this study employed a scientific research method and conducted an empirical analysis through the following steps: first, questionnaires were developed based on established measurement scales, and data were collected through distribution and retrieval. Second, confirmatory factor analyses (CFAs) were performed using AMOS 26.0 to examine the discriminant validity of key variables, followed by tests for common method bias and data aggregation analysis to ensure the study's reliability and validity. Third, structural equation modeling (SEM) was conducted using AMOS 26.0 to test the hypothesized relationships among variables and to evaluate the overall fit of the theoretical model. Finally, SPSS 29.0 was utilized for descriptive statistical analysis, correlation analysis, and multiple regression analysis to provide supplementary hypothesis testing and assess the robustness of the results.

### 3.1 Sample and procedure

This study adopts a quantitative, cross-sectional design to investigate the relationships between multitasking, workplace wellbeing, job stress, and job autonomy. The target population consisted of full-time employees of information technology (IT) industry in China.

In this study, all ethical standards for human subject research were strictly followed. Participation was entirely voluntary and anonymous. Prior to starting the survey, each participant was presented with an online informed consent page that explained the purpose of the study, the voluntary nature of participation, and the confidentiality of responses. Only participants who gave explicit consent by clicking “Agree” could proceed to the questionnaire. All participants were adult full-time employees in the IT industry with at least 6 months of related work experience. No identifying information was collected, and data were used solely for academic research and stored securely with restricted access.

The survey items were uploaded and hosted on Wenjuanxing (https://www.wjx.cn), a widely used online survey platform in China. The questionnaire link was then manually distributed by the researchers to target respondents working in the IT industry via professional and social networks. A snowball sampling strategy was employed: initial participants were invited to complete the survey and were encouraged to forward the link to suitable colleagues within their organizations, thereby expanding the reach and diversity of the sample. To encourage participation, respondents who completed the survey received a small incentive in the form of a three CNY digital voucher, which was distributed through anonymous online means in accordance with ethical standards and confidentiality requirements.

360 IT employees who meet the survey criteria were invited to participate this research. While the high response rate reflects the effectiveness of the trust-based recruitment and incentive approach, we acknowledge that the use of snowball sampling may introduce selection bias and limit generalizability. This limitation is discussed further in the final section of the paper.

To evaluate whether the sample size was sufficient for structural equation modeling (SEM), a minimum sample size of 200 to ensure stable parameter estimates and a reliable model fit (Jackson, 2003; Wolf et al., 2013). With a final sample of 354 participants, this study surpassed this benchmark, indicating that the sample size is adequate to provide sufficient statistical power for identifying significant relationships among the variables examined.

### 3.2 Demographic information

In this study, a total of 360 questionnaires were distributed, of which 358 were successfully retrieved, resulting in a response rate of 99.44%. After eliminating invalid responses due to duplication, incompleteness, or failure to meet the required criteria, 354 valid questionnaires remained, yielding a valid response rate of 98.33%. The descriptive statistics for the final sample utilized in the formal survey are presented in Table 1.

**Table 1:** The descriptive statistics of samples for the formal surveys.

**Characteristic**	**Classification**	**Amount**	**Ratio %**
Gender	Male	182	51.4
	Female	172	48.6
Age group	≤25 years old	40	11.3
	26–30 years old	73	20.6
	31–40 years old	138	39.0
	41–50 years old	75	21.2
	≥51 years old	28	7.9
Education level	Diploma	107	30.2
	Bachelor	220	62.1
	Master or above	27	7.6
Tenure	≤2 years	56	15.8
	3–5 years	80	22.6
	6–10 years	125	35.3
	≥11 years	93	26.3

The demographic data presented in Table 1 indicate that the sample comprised 51.4% male and 48.6% female respondents. In terms of age distribution, 11.3% were 25 years old or younger, 20.6% were between 26 and 30 years old, 39.0% were between 31 and 40 years old, 21.9% were between 41 and 50 years old, and 7.9% were 51 years old or above. Regarding educational attainment, 30.2% had a diploma, 62.1% held a bachelor's degree, and 7.6% possessed a master's degree or higher. In terms of job tenure, 15.8% had 2 years of work experience or less, 22.6% had 3 to 5 years of work experience, 35.3% had 6 to 10 years of experience, and the remaining 26.3% had 11 years of work experience or more.

To assess the representativeness of the sample, we compared the demographic profile of respondents with publicly available statistics on China's workforce in IT industry. An increasing presence of women in IT-related roles. The concentration of respondents between 31 and 40 years of age and the predominance of bachelor's degrees also reflect broader national patterns. These similarities suggest that the sample is reasonably representative of the target population, although selection bias cannot be fully ruled out.

### 3.3 Measures

The measurement scales in this study (Table 2) were adopted from widely recognized instruments developed by both domestic and international scholars. All scales have demonstrated strong reliability and content validity in previous research and are commonly cited in leading academic journals. This approach ensured the use of valid, standardized measures for each variable.

**Table 2:** Measurement scales for main study variables.

**Construct**	**Source**	**Number of items**	**Sample item**	**Cronbach's α**
Multitasking	Ji and Xu (2023)	3	“I have to balance several projects at once.”	0.807
Job stress	Parker and DeCotiis (1983)	13	“I have felt fidgety or nervous as a result of my job”	0.947
Workplace wellbeing	Zheng et al. (2015)	6	“I am satisfied with my work responsibilities.”	0.909
Job autonomy	Morgeson and Humphrey (2006)	9	“The job allows me to make my own decisions about how to schedule my work.”	0.933

Multitasking was assessed using a five-point Likert scale ranging from 1 (“never”) to 5 (“always”). Higher scores indicate more frequent multitasking demands. Meanwhile, the other three variables—Job Stress, Workplace Wellbeing, and Job Autonomy—were measured using a five-point Likert scale ranging from 1 (“totally disagree”) to 5 (“totally agree”).

Multitasking. The three-item scale adopted from Ji and Xu (2023) was used to measure multitasking. In this scale, two items were selected from the challenging stressor scale of LePine et al. (2016) and one item was selected from the study of Kapadia and Melwani (2021) to form this 3-item scale (Ji and Xu, 2023). Sample items include “I have to balance several projects at once.” and “I have to multitasking my assigned projects.” (α = 0.807).

Job stress. The 13-item scale was developed by Parker and DeCotiis (1983) used to measure job stress. Sample items include “I have felt fidgety or nervous as a result of my job” and “Working here makes it hard to spend enough time with my family.” (α = 0.947).

Workplace wellbeing. The six-item scale was developed by Zheng et al. (2015) used to measure workplace wellbeing. The scale is consistent with research on Chinese scenarios. Sample items include “I am satisfied with my work responsibilities” and “In general, I feel fairly satisfied with my present job.” (α = 0.909).

Job autonomy. The nine-item scale developed by Morgeson and Humphrey (2006) was used to measure job autonomy. Sample items include “The job allows me to make my own decisions about how to schedule my work” and “The job allows me to decide on the order in which things are done on the job.” (α = 0.933).

#### 3.3.1 Control variables

This study controlled for gender, age, education, and tenure. These demographic factors can influence how employees perceive job demands and resources, and affect their ability to manage multitasking, stress, and autonomy (Becker et al., 2023; Kirchberg et al., 2015; Mark et al., 2014; Zacher and Schmitt, 2016). Multitasking performance has been shown to vary by gender and age, with females making fewer errors, while both younger and older participants took longer to complete multitasking tasks—suggesting an inverted U-shaped pattern where efficiency peaks in mid-age (Crews and Russ, 2020). Controlling for these variables helps isolate the unique relationship of the core variables.

### 3.4 Results

#### 3.4.1 Confirmatory factor analysis

**Figure 2:**
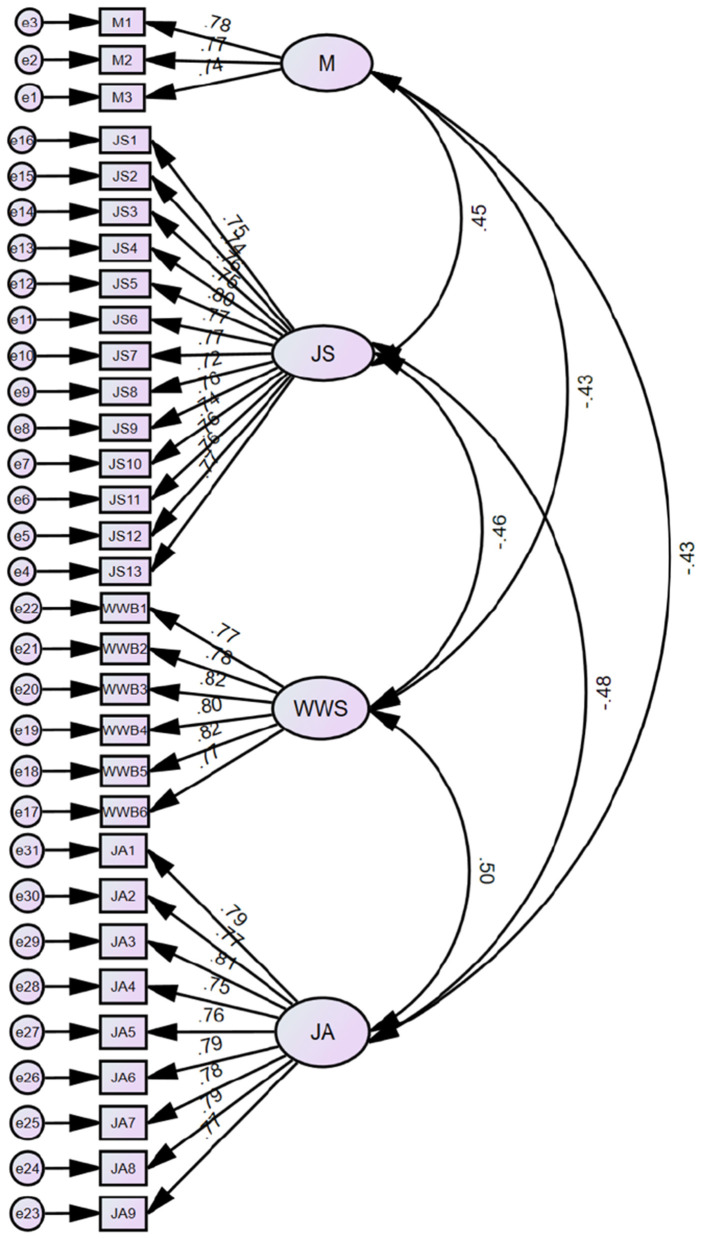
Structural equation model. M, multitasking; JS, job stress; WWB, workplace wellbeing; JA, job autonomy.

Based on the conceptual framework, a structural equation model was constructed, including four main variables and 31 measurement items (Figure 2). Prior to conducting discriminant and convergent validity tests, a factor analysis of the measurement items was performed using SPSS 29.0. The results indicated a highly significant probability (*p* < 0.001) and a Kaiser-Meyer-Olkin (KMO) index of 0.956, confirming the suitability of the data for exploratory factor analysis.

Furthermore, composite reliability (CR) was assessed for each variable, with all factor loadings exceeding 0.8 and the average variance extracted (AVE) values surpassing 0.5, demonstrating satisfactory convergent validity. To assess discriminant validity among the variables, this study employed AMOS 26.0 to conduct confirmatory factor analysis (CFA) on four constructs: multitasking, job stress, workplace wellbeing, and job autonomy.

#### 3.4.2 Assessment of common method bias

As the data for this study were collected through employee self-reports, there is a potential risk of common method bias. To assess potential common method bias (CMB), two complementary approaches were employed. First, to assess this, Harman's single-factor test was conducted using exploratory factor analysis. The results revealed that four factors accounted for 65.042% of the total variance, with the first factor explaining 39.270%, which is below the critical threshold of 50%. Since no single factor accounted for the majority of the variance, these findings indicate that common method bias was not a significant concern in this study.

Additionally, based on the results of the confirmatory factor analysis for common method bias as suggested by Podsakoff et al. (2003) (see Table 3), the one-factor model demonstrated poor fit indices (χ^2^/df = 7.135, TLI = 0.582, CFI = 0.609, RMSEA = 0.132, RMR = 0.179), falling below the minimum acceptable thresholds. In contrast, the four-factor model showed a significantly better fit (χ^2^/df = 1.101, TLI = 0.993, CFI = 0.994, RMSEA = 0.017, RMR = 0.039). The comparison between the two models revealed a statistically significant difference (Δχ^2^ = 2,625.398, Δdf = 6, *p* < 0.001). These results indicate that common method bias is not a serious concern in this study.

**Table 3:** Results of confirmatory factor analysis.

**Model**	**χ^2^**	**df**	**χ^2^/df**	**TLI**	**CFI**	**GFI**	**NFI**	**IFI**	**RMSEA**	**RMR**
Four-factor model	471.158	428	1.101	0.993	0.994	0.924	0.935	0.994	0.017	0.039
Three-factor model	767.052	431	1.780	0.947	0.951	0.872	0.895	0.951	0.047	0.076
Two-factor model	1,744.257	433	4.028	0.793	0.808	0.666	0.760	0.809	0.093	0.139
Single-factor model	3,096.556	434	7.135	0.582	0.609	0.442	0.575	0.611	0.132	0.179

Four-factor model: M, JS, WWB, JA; three-factor model: M+JS, WWB, JA; two-factor model: M+JS+WWB, JA; single-factor model: M+JS+WWB+JA.

M, multitasking; JS, job stress; WWB, workplace wellbeing; JA, job autonomy.

#### 3.4.3 Descriptive analyses

The means, standard deviations, and correlation coefficients among the study variables are presented in Table 4. As shown in Table 4, multitasking exhibited a significant positive correlation with job stress (*r* = 0.394, *p* < 0.01) and a significant negative correlation with workplace wellbeing (*r* = −0.368, *p* < 0.01). Additionally, job stress was negatively correlated with workplace wellbeing (*r* = −0.427, *p* < 0.01).

**Table 4:** Means, standard deviations, and correlations.

**Variable**	**M**	**SD**	**M**	**JS**	**WWB**	**JA**
M	3.275	0.986	1			
JS	3.314	0.913	0.394^**^	1		
WWB	2.577	0.970	−0.368^**^	−0.427^**^	1	
JA	2.661	0.939	−0.371^**^	−0.453^**^	0.463^**^	1

^*^p < 0.05; ^**^p < 0.01.

M, Multitasking; JS, job stress; WWB, workplace wellbeing; JA, job autonomy.

Furthermore, a variance inflation factor (VIF) analysis was conducted to assess potential multicollinearity in the regression model. The results indicated that the maximum VIF value for any variable in the model was 1.359, which is well below the commonly accepted threshold of 10. This suggests that multicollinearity was not a significant concern in this study.

Overall, these findings provide initial support for the validity of the proposed variables and offer preliminary evidence for hypothesis testing.

#### 3.4.4 Hypothesis testing

Regression analysis is a statistical method used to examine the quantitative relationships between two or more variables. It serves as a predictive modeling technique that involves analyzing the specific nature of the correlations among variables and identifying any causal relationships (De Veaux et al., 2016). In this study, hierarchical regression analysis was conducted using SPSS 29.0 to test the proposed hypotheses. Additionally, the mediating role of job stress in the relationship between multitasking and workplace wellbeing is assessed using the causal steps approach. The findings are presented in Table 5.

**Table 5:** The multiple regression analysis: the mediating effect of job stress.

**Variable**	**Dependent variable: workplace wellbeing**		
	**Model 1**	**Model 2**	**Model 3**
**Control variable**			
Gender	0.000	−0.017	−0.007
Age	−0.070	−0.018	−0.004
Education level	0.111^*^	0.120^*^	0.107^*^
Tenure	0.087	0.053	0.064
**Independent variable**			
Multitasking		−0.374, *p* < 0.001	−0.243, *p* < 0.001
**Mediating variable**			
Job stress			−0.334, *p* < 0.001
*R^2^*	0.013	0.152	0.246
Adjusted *R^2^*	0.002	0.140	0.233
ΔR^2^	0.013	0.139	0.094
*F*	1.165	12.480	18.831
ΔF	1.165	56.990, *p* < 0.001	43.046, *p* < 0.001

^*^p < 0.05, ^**^p < 0.01, *p* < 0.001.

As shown in Model 2 in Table 5, after controlling for the effects of gender, age group, education, tenure, multitasking was significantly negative related to workplace wellbeing (β = −0.374, *p* < 0.001), so hypothesis 1 was supported.

Multitasking has a significant positive relationship on job stress (β = 0.393, *p* < 0.001), as shown in Model 5 in Table 6. Model 3 in Table 5 shows that job stress has a significant negative association on workplace wellbeing (β = −0.334, *p* < 0.001). Multitasking has a significant negative impact on workplace wellbeing (β = −0.243, *p* < 0.001), as shown in Model 3 in Table 5. Therefore, job stress plays as a partial mediator on the relationship between multitasking and workplace wellbeing. Hypothesis 2 was supported. The authors used the Process v3.5 by Andrew F. Hayes to double check the mediation role of job stress, it showed the same outcome (as shown in Table 7), therefore, Hypothesis 2 was supported.

**Table 6:** The multiple regression analysis: the moderating effect of job autonomy.

**Variable**	**Job stress**			
	**Model 4**	**Model 5**	**Model 6**	**Model 7**
**Control variable**				
Gender	0.012	0.031	−0.004	−0.012
Age	0.097	0.042	0.009	0.040
Education	−0.029	−0.038	−0.034	−0.023
Tenure	−0.003	0.032	0.036	0.018
**Independent variable**				
Multitasking		0.393, *p* < 0.001	0.262, *p* < 0.001	0.907, *p* < 0.001
**Moderating variable**				
Job autonomy			−0.353, *p* < 0.001	0.483^**^
**Product term**				
Multitasking × job autonomy				−0.878, *p* < 0.001
*R^2^*	0.009	0.162	0.267	0.321
Adjusted *R^2^*	−0.002	0.150	0.255	0.307
ΔR^2^	0.009	0.153	0.105	0.054
*F*	0.794	13.441, *p* < 0.001	21.091, *p* < 0.001	23.339, *p* < 0.001
ΔF	0.794	63.463, *p* < 0.001	49.896, p < 0.001	27.257, *p* < 0.001

^*^p < 0.05, ^**^p < 0.01, *p* < 0.001.

**Table 7:** Mediation analysis, job stress as a mediator.

**Exogenous variable**	**Direct effect**	**Indirect effect**	**Total effect**	**VAF range**	**Mediation**	**Endogenous variable**
Multitasking	−0.2326	−0.1293	−0.3619	0.3573	Partial	Workplace wellbeing

To test the moderating effect of job autonomy between multitasking and job stress, Model 7 introduces the product terms of multitasking and job stress based on Model 6, as shown in Table 6.

The operation result of the Model 7 shows that the product terms of multitasking and job stress are significantly related to job stress (β = −0.878, *p* < 0.001), suggesting that the job autonomy negatively moderates the relationship between multitasking and job stress. Therefore, Hypothesis 3 receives initial support.

**Figure 3:**
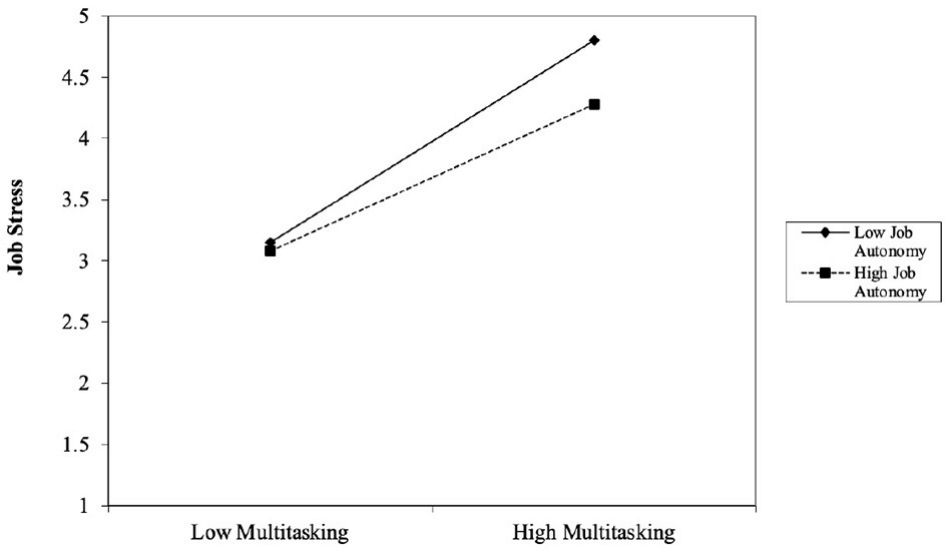
Moderating effect of JA.

To further validate this moderating effect, job autonomy was categorized into high and low levels, represented as one standard deviation above and below the mean, respectively. The impact of multitasking on job stress was then examined under these different conditions. The specific results are illustrated in Figure 3.

As illustrated in Figure 3, job stress significantly increases as multitasking levels rise, regardless of the degree of job autonomy. However, under conditions of low job autonomy (solid line), the increase in job stress is more pronounced, indicating that when employees have limited autonomy in their work, the negative impact of multitasking on job stress is amplified. In contrast, under high job autonomy (dashed line), job stress also increases with multitasking but at a slower rate, suggesting that greater job autonomy can help mitigate the adverse links of multitasking on job stress.

These findings indicate that while multitasking contributes to heightened job stress, higher levels of job autonomy serve as a buffering mechanism, alleviating its negative impact. Therefore, the results provide further empirical support for Hypothesis 3.

## 5 Discussion

This study explored the impact of multitasking on workplace wellbeing, the mediating role of job stress in the relationship between multitasking and workplace wellbeing, and the moderating role of job autonomy in the link between multitasking and job stress. The findings offered valuable insights into these complex interactions. The results from the mediation analysis revealed that job stress partially mediates the connection between multitasking and workplace wellbeing. Additionally, the moderation analysis showed that job autonomy negatively moderates the relationship between multitasking and job stress.

This study confirmed that multitasking has a negative association with employee's workplace wellbeing. It is consistent with Syndicus and Wiese (2024), which showed that office environments multitasking is prevalent have shown decreased affective wellbeing. The existing research on the pathways of multitasking on workplace wellbeing is scarce. The results indicate that job stress partially mediates the negative impact of multitasking on workplace wellbeing, suggesting that while multitasking itself may not entirely determine an employee's workplace wellbeing, the stress induced by multitasking plays a critical role in diminishing workplace wellbeing. This aligns with existing research indicating that multitasking is positively associated with stress (Ghazanfar and Jumani, 2021; Mark et al., 2014) and workplace stressors significantly contribute to decreased employee wellbeing (Jex et al., 2014). However, the existing research link multitasking to workplace wellbeing with the mediating role of job stress is scarce.

Moreover, while the results indicate that job stress partially mediates the relationship between multitasking and workplace wellbeing (VAF = 35.73%), a large proportion of the variance remains unexplained. This suggests the presence of other possible mediating mechanisms. Variables such as emotional exhaustion, role conflict, cognitive overload, or reduced self-efficacy may also play important roles in linking multitasking to wellbeing. Future studies are encouraged to adopt a broader framework incorporating multiple mediators to better understand the complexity of this relationship.

Although the observed correlation between multitasking and job stress (*r* = 0.39) is moderate in magnitude, it holds meaningful practical significance in organizational contexts. According to Cohen's (1988) benchmarks, an r of 0.30 to 0.50 indicates a moderate effect size, which, in real-world settings—particularly within high-demand sectors such as IT—can translate into substantial impacts on employees' stress levels, job satisfaction, and health outcomes. Even small to moderate increases in job stress can lead to higher absenteeism, burnout, and reduced productivity over time. Therefore, interventions aimed at managing multitasking demands, even if modestly effective, may yield valuable benefits for workforce wellbeing.

Furthermore, this study identifies job autonomy as a negative moderator in the relationship between multitasking and job stress, meaning that higher job autonomy weakens the adverse link of multitasking to job stress levels. Employees with greater control over their tasks and workflows appear better equipped to manage the stressors associated with multitasking, likely because autonomy fosters problem-solving flexibility, reduces perceived helplessness, and enables individuals to prioritize tasks strategically.

Job autonomy serves as a buffer against workplace stress, providing employees with greater control over their tasks and reducing the detrimental effects of workplace demands (Wan et al., 2024). However, other studies have shown that excessive job autonomy can sometimes lead to increased work pressure, particularly in remote work settings (Saragih et al., 2021). These nuanced findings suggest that while job autonomy can mitigate stress, organizations should carefully balance autonomy with clear support structures to optimize employee wellbeing.

Although the moderation effect of job autonomy was statistically significant, its effect size was modest. This suggests that while job autonomy plays a buffering role in the relationship between multitasking and job stress, it may not be sufficient on its own to offset the adverse impacts of high multitasking demands. Other organizational and personal resources—such as managerial support, time management skills, or task prioritization—might also be needed to more effectively manage the stress associated with multitasking. Future research could explore these additional moderators to better understand how to enhance employee wellbeing in high-demand work environments.

While the control-only model (Model 4) yielded a low R^2^ value (0.009), this is not unexpected given that demographic variables often have limited explanatory power in organizational behavior research. Subsequent models including multitasking, job stress, and job autonomy improved the explanatory capacity (*R*^2^ = 0.321). While modest in magnitude, we note that such effect sizes are typical in organizational and psychological research, especially in complex, real-world environments. In real workplaces, even small associations can have meaningful practical implications when accumulated over time or across large employee populations. Still, the unexplained variance suggests that future research should consider additional psychological or contextual variables to enhance predictive accuracy.

The findings reflect a broader cultural-organizational context in China that shapes the interaction between multitasking, job stress, job autonomy, and workplace wellbeing. First, Chinese workplace culture, rooted in collectivism and Confucian values, emphasizes group harmony, hierarchy, and face-saving. Employees may feel pressured to comply and multitask to avoid losing face, increasing stress and weakening the stress-buffering role of autonomy. Second, the “996” work culture common in China's IT sector intensifies stress from multitasking but may also enhance the value of autonomy, as workers seek control amid demanding schedules. Third, due to high power distance, autonomy in China is often expected to align with managerial guidance. This may explain the limited moderating effect observed—autonomy is more effective when socially sanctioned rather than fully self-directed. Overall, while autonomy can buffer stress, its impact is shaped by cultural norms. Practical strategies should adapt accordingly, such as promoting autonomy in non-face-threatening ways and allowing discretion within clear hierarchical structures.

In addition, although the JD-R and COR theory framework guided the hypotheses, other theoretical models may provide valuable alternative explanations for our findings: Transactional Stress Theory (Lazarus and Folkman, 1984) focuses on how individuals appraise stressors and utilize coping strategies. Multitasking stress may partly reflect how employees interpret and cope with demands, rather than demand exposure alone. Self-Determination Theory (SDT) emphasizes basic psychological needs—autonomy, competence, and relatedness—as drivers of wellbeing. Job autonomy likely alleviates stress by satisfying these needs and supporting autonomous motivation, which in turn fosters wellbeing in work environments. Effort-Reward Imbalance (ERI) theory suggests that stress arises when high effort (e.g., multitasking) is met with insufficient rewards. Even with autonomy, if rewards do not match efforts, employees may remain stressed. By integrating or comparing these alternative models, future research can uncover whether moderating factors like appraisal (Transactional), motivational dynamics (SDT), or reward perceptions (ERI) better explain the multitasking–stress–wellbeing link.

### 5.1 Theoretical implications

This study contributes to the growing literature on workplace wellbeing by demonstrating the dual role of job stress as a mediator and job autonomy as a moderator in the relationship of multitasking and wellbeing. These findings support and extend the Job Demand-Resource (JD-R) theory, emphasizing that while multitasking increases job demands, resources such as job autonomy can help mitigate negative outcomes (Sharma and Sharma, 2024). Additionally, our results align with Conservation of Resources (COR) theory, suggesting that employees who lack autonomy may experience heightened job stress, leading to reduced wellbeing (Islam and Alam, 2024). Future research could explore how other contextual factors, such as workplace culture and leadership styles, further influence these relationships.

While grounded in JD-R and COR, this study did not evaluate alternative explanatory frameworks such as Transactional Stress Theory, Self-Determination Theory, and Effort-Reward Imbalance models. Future research should test these competing approaches, for example by measuring stress appraisals, basic need satisfaction, and relative reward perceptions, to determine which mechanisms most strongly mediate the multitasking–stress and multitasking–wellbeing relationships.

### 5.2 Practical implications

From a managerial perspective, these findings underscore the importance of balancing multitasking demands with adequate workplace resources. There are several aspects that organizations should consider.

Employers should ensure that employees are not overwhelmed with multitasking responsibilities, as excessive cognitive load can impair wellbeing and productivity. Providing task prioritization strategies and structured work schedules can help mitigate stress. Moreover, to better support employees working under high multitasking and overtime demands managers should grant autonomy across specific dimensions: work methods autonomy (letting employees choose tools and strategies), work scheduling autonomy (providing flexibility in task timing and breaks), and decision-making autonomy (empowering employees to prioritize and sequence tasks). These practices have been linked to enhanced engagement, stress buffering, and wellbeing among Chinese employees in high-demand environments. Furthermore, organizations should integrate stress management interventions, such as mindfulness training and employee assistance programs, to help employees cope with job stress. In addition, creating a workplace culture that values employee wellbeing, encourages breaks, and fosters open communication can further buffer against the negative effects of multitasking.

The moderation analysis showed that job autonomy weakened the correlation between multitasking and job stress, though the effect size was moderate. To translate this finding into practice, organizations should consider redesigning job roles to enhance decision-making latitude. For instance, enabling employees to choose the sequence of tasks or set their own deadlines can amplify autonomy and better buffer multitasking pressures. This recommendation aligns with the foundational premise of the demand-control model, which highlights decision latitude as a key resource under high-demand conditions.

However, because the buffering role of autonomy was modest, employers should also incorporate additional supports. Studies have shown that managerial support can further reduce job strain when autonomy alone is insufficient. Offering structured time-management training, mentorship programs, and flexible scheduling can help employees use their autonomy more effectively amid multitasking demands.

Finally, organizations may benefit from periodically monitoring employees' stress levels and work patterns. Simple measures—such as brief surveys or one-on-one check-ins—can identify individuals who are not benefiting fully from autonomy and allow targeted interventions, such as adjusting workload or offering coaching in task prioritization.

### 5.3 Limitations and directions for future research

Despite its contributions, this study has several limitations that warrant consideration. First, this study's reliance on a homogeneous sample of full-time R&D employees from IT companies in China, recruited through snowball sampling within a specific professional network, limits the generalizability of the findings. The non-random sampling method may introduce selection bias, potentially overrepresenting certain job roles, company cultures, or stress coping styles. Although the IT sector was chosen due to the high prevalence of multitasking, organizational structures, and work norms vary widely across regions and industries. Future research should enhance external validity by incorporating more diverse and representative samples across different sectors and geographic areas, such as financial services, e-commerce, or high-tech manufacturing.

Second, the cross-sectional design prevents causal inference. While the study was theoretically grounded in the Job Demands-Resources (JD-R) and Conservation of Resources (COR) frameworks, which imply directional relationships, the data only support correlational interpretations. Longitudinal or experimental designs would allow future researchers to assess the temporal ordering and potential causality of these relationships.

Third, all variables in this study were measured through self-reports from a single source, which raises concerns about potential self-report bias. For instance, multitasking was assessed via self-reported measures that may be affected by individual perception or recall inaccuracies. While the scale used demonstrated acceptable internal consistency, it may not accurately capture real-time task-switching behavior. Although statistical tests indicated that common method bias was not a severe threat, factors such as social desirability, memory limitations, or perceptual bias may still have influenced the results. Future research could enhance robustness by incorporating multi-source or behavioral data, such as supervisor ratings or performance logs.

Several methodological limitations should be acknowledged. First, the measurement of multitasking was based on a three-item scale, which—although exhibiting acceptable internal consistency (α = 0.807)—may not fully capture the complexity and multidimensionality of multitasking in the workplace. Multitasking involves not only task switching but also simultaneous task processing and cognitive load management, which may not be fully represented with a brief scale. Future research is encouraged to adopt more comprehensive or validated multidimensional instruments to better reflect the construct in diverse work contexts.

Furthermore, although the study achieved a very high valid response rate (98.33%), a formal non-response bias assessment was not conducted due to the absence of data from non-respondents. While the relatively low dropout rate suggests minimal risk, the possibility of systematic differences between respondents and non-respondents cannot be entirely ruled out. Subsequent research could mitigate this limitation by incorporating follow-up with non-respondents or applying statistical imputation techniques.

Additionally, an inspection of the descriptive statistics indicated that no clear ceiling or floor effects were present in the key variables—multitasking, job stress, job autonomy, and workplace wellbeing—as the means and standard deviations fell within moderate ranges on their respective Likert-type scales. Nevertheless, future studies should remain vigilant regarding potential range restriction, especially in self-report data, where response clustering at scale extremes can attenuate correlations or compromise measurement sensitivity.

## Data Availability

The raw data supporting the conclusions of this article will be made available by the authors, without undue reservation.

## Appendix

**Appendix survey instrument Multitasking (Ji and Xu 2023)**

1. I have to balance several projects at once.

2. I have to multitasking my assigned projects.

3. My job requires me to do two or more tasks at once.

**Workplace wellbeing (WWB) (Zheng et al., 2015)**

1. I am satisfied with my work responsibilities.

2. In general, I feel fairly satisfied with my present job.

3. I find real enjoyment in my work.

4. I can always find ways to enrich my work.

5. Work is a meaningful experience for me.

6. I feel basically satisfied with my work achievements in my current job.

**Job stress scale (Parker and DeCotiis, 1983)**

1. I have felt fidgety or nervous as a result of my job.

2. Working here makes it hard to spend enough time with my family.

3. My job gets to me more than it should.

4. I spend so much time at work, I can't see the forest for the trees.

5. There are lots of times when my job drives me right up the wall.

6. Working here leaves little time for other activities.

7. Sometimes when I think about my job I get a tight feeling in my chest.

8. I frequently get the feeling I am married to the company.

9. I have too much work and too little time to do it in.

10. I feel guilty when I take time off from job.

11. I sometimes dread the telephone ringing at home because the call might be job-related.

12. I feel like I never have a day off.

13. Too many people at my level in the company get burned out by job demands.

**Job autonomy (Morgeson and Humphrey, 2006) Work scheduling autonomy**

1. The job allows me to make my own decisions about how to schedule my work.

2. The job allows me to decide on the order in which things are done on the job.

3. The job allows me to plan how I do my work.

**Decision-making autonomy**

1. The job gives me a chance to use my personal initiative or judgment in carrying out the work.

2. The job allows me to make a lot of decisions on my own.

3. The job provides me with significant autonomy in making decisions.

**Work methods autonomy**

1. The job allows me to make decisions about what methods I use to complete my work.

2. The job gives me considerable opportunity for independence and freedom in how I do the work.

3. The job allows me to decide on my own how to go about doing my work.
